# Supplementary material for: Use of Glycoproteins—Prostate-Specific Membrane Antigen and Galectin-3 as Primary Tumor Markers and Therapeutic Targets in the Management of Metastatic Prostate Cancer
Source: Cancers (Basel). 2022 May 30;14(11):2704. doi: 10.3390/cancers14112704 (PMC9179331; doi:10.3390/cancers14112704)
Supplement: Supplementary file 1 [file cancers-14-02704-s001.zip › cancers-1655430-supplementary.pdf]

Supplementary Materials: Use of Glycoproteins—Prostate-Specific Membrane Antigen and Galectin-3 as Primary Tumor Markers and Therapeutic Targets in the Management of Metastatic Prostate Cancer

Satish Sharma, Katherine Cwiklinski, Donald E Sykes, Supriya D. Mahajan, Kent Chevli, Stanley A. Schwartz and Ravikumar Aalinkeel

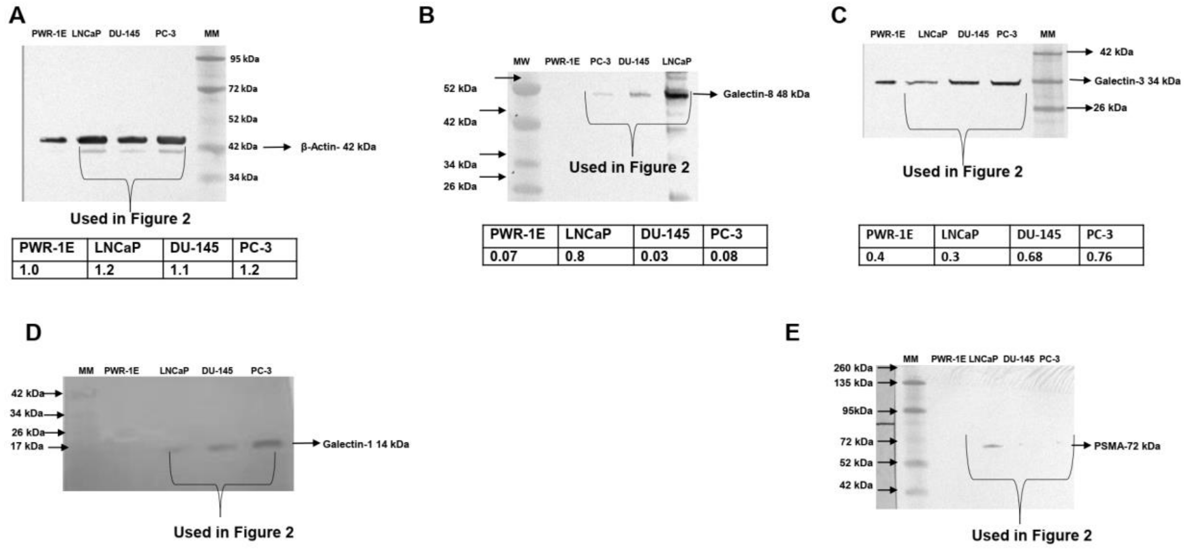

A

PWR-1E	LNCaP	DU-145	PC-3
1.0	1.2	1.1	1.2

B

PWR-1E	LNCaP	DU-145	PC-3
0.07	0.8	0.03	0.08

C

PWR-1E	LNCaP	DU-145	PC-3
0.4	0.3	0.68	0.76

D

PWR-1E	LNCaP	DU-145	PC-3
0.01	0.02	0.15	0.32

E

PWR-1E	LNCaP	DU-145	PC-3
0.01	0.35	0.03	0.02

Figure S1. Original Western blots, Western blot analysis showing expression of β-Actin (**A**), Galectin-8(**B**), Galectin-3 (**C**), Galectin-1(**D**) and PSMA (**E**) in in PWR-1E, LNCaP, DU-145 and PC-3. Prepared cell pellets resuspended in lysis buffer [65 mmol/Tris–HCl (pH 7·4), 150 mmol/L NaCl, 1 mmol/L EDTA, 1% nonidet-P40, 1% sodium deoxycholate, 1 μg/mL aprotinin, 100 μg/mL PMSF, for 30 min at 4 and cleared by centrifugation for 30 min at 13,000× g. Supernates collected fractionated by 10% SDS–PAGE, transferred to nitrocellulose membranes, incubated with primary antibody to Gal-8, Gal-3, Gal-1 and PSMA as indicated in the methods section followed by horseradish peroxidase-conjugated secondary antibodies, and revealed with Super Signal Pico West (Pierce, Rockford, IL). β-Actin expression was used as an internal control.
